# Aortic Stenosis Prevention: Is a New Cardiovascular Disease Paradigm Coming of Age?

**DOI:** 10.3390/jcm14030903

**Published:** 2025-01-29

**Authors:** Antonios Halapas, Dennis V. Cokkinos

**Affiliations:** 1 Department of Interventional Cardiologist and THV Program, Athens Medical Center, 11526 Athens, Greece; 2 Clinical, Translational and Experimental Surgery Research Centre, Biomedical Research Foundation Academy of Athens, 4, Soranou Ephessiou Str., 11527 Athens, Greece; dcokkinos@bioacademy.gr

**Keywords:** aortic stenosis, pathophysiology, diagnosis, prevention, management

## Abstract

Calcific aortic stenosis (CAS) is currently recognized as the third most frequent cardiovascular disorder in persons aged above 60 years, after atherosclerotic disease and hypertension, and together with its precursor aortic sclerosis it has been found in more than 30% of elderly individuals. CAS is an active multifactorial process characterized by a progressive fibro-calcific remodeling and thickening of the AV leaflets caused by hemodynamic flow factors, genetic factors, lipoprotein deposition, oxidation, chronic inflammation, immunomodulators, and finally osteoblastic transformation of cardiac. Herein a comprehensive state-of-the-art paper is presented regarding the underlying pathophysiological mechanisms of CAS and the potential preventive strategies as an alternative to surgical and interventional treatment.

## 1. Introduction—The Problem of CAS

CAS is currently recognized as the third most frequent cardiovascular disease (CVD) in persons aged above 60 years, after atherosclerosis and hypertension. Together with its precursor aortic sclerosis, it has been found in more than 30% of elderly individuals. Although rheumatic valvulopathy prevalence has been drastically diminished in developed countries, the global burden of CAS is increasing due to ageing of the population, accounting for 34% of all native valve disease and 43% of all single valve disease. Additionally, epidemiological data describe an exponential increase in the prevalence of CAS with age i.e., 0.2% in the 50–59-year group, 1.3% in the 60–69-year group, 3.9% in of the 70–79-year group, and 9.8% in those aged 80–89 years [1]. The incidence of newly diagnosed AS is increasing, reaching 5 per 1000 per year, with the initial mean age being 60 years [2]. Our team has been involved in translating research evidence into clinical practice, thus efforts towards CAS prevention or regression may start from: general population, CAS patients, and valve studies.

## 2. Pathophysiology Mechanisms—Risk Factors of Calcific AS (CAS)

The aortic valve (AV) is structured to withstand a wide range of stresses during the cardiac cycle, both hemodynamic and mechanical. A normal AV is composed of three tissue layers, each <1 mm thick. On the aortic side lies the fibrosa or aortic layer, rich in fibroblasts and collagen fibers arranged circumferentially. On the ventricular side of the leaflet lies the ventricularis, abundant in elastin and collagen fibers aligned in a radial direction, providing flexibility. The spongiosa between these two layers is composed of loosely arranged layers of connective tissue containing fibroblasts, elastic fibers, mesenchymal cells and a mucopolysaccharide-rich matrix acting as a lubricant, diminishing the friction between the two external layers, providing flexibility. Within AV cusps, two types of cells can be distinguished, valve endothelial cells (VECs) and valve interstitial cells (VICs), which are responsible for the valvular structure and function maintenance. VECs are present on both sides of the cusp, while VICs are located in the extracellular matrix (ECM) filling the body of the AV. VICs are the predominant cells within the valve and as a heterogeneous population include fibroblasts, myofibroblasts, and smooth muscle cells [3]. Reduced numbers of VICs due to apoptosis are associated with increased risk of CAS development [3]. Additionally, mechano-sensitive channels trigger osteogenic VIC transformation, producing a thick and rigid ECM [4].

**Figure 1:**
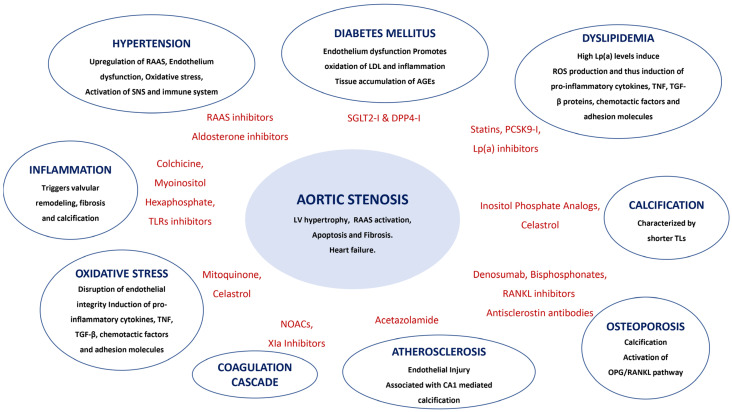
Knowledge of underlying pathophysiological mechanisms of calcific aortic stenosis development will support potential preventive treatment strategies.

CAS is a multifactorial process characterized by a progressive fibro-calcific remodeling and thickening of the AV leaflets caused by hemodynamic flow factors, genetic factors, lipoprotein deposition, oxidation, chronic inflammation, immunomodulators, and osteoblastic transformation of cardiac VICs (Figure 1). The repeated opening and closing of AV cusps during a cardiac cycle increases mechanical stress disrupting the structural stability of the endothelial layer. Due to the mechano-transduction process, once valve sclerosis develops, flow disturbance is exacerbated further affecting the aforementioned degeneration [5]. Thus, stenosis begets stenosis. This sets the stage for the therapeutic interventions to be further described. Liu et al. stress that the murine transverse aortic constriction model leads to activation of many immune cell sub-population, i.e., mast cells, monocytes, macrophages, neutrophils, dendritic cells, eosinophils, and natural killer cells [3]. Steady laminar flow can be atheroprotective, while disturbed flow is athero-prone, through mechanisms such as: Kruppel-like factor 2 and 4, PPARs, e-NOS, VACAM-1, ICAM1 and E-selectin [5]. Disruption or malfunction of the endothelial layer covering the fibrosa induces the uptake of oxidatively modified lipids (such as LP(a)), red blood cells, and immune cells, all of which trigger an inflammation process and valvular remodeling favoring fibrosis and VIC calcification. Moreover, two more mechanisms, important for comprehending the pathophysiology of the CAS process but also offering therapeutic perspectives, should be noted: (a) the complement activation, falling into the general immune activation pattern. Apart from specific complement inactivating already being used clinically, both SGLT2 and PCSK9 inhibitors demonstrate such an action, and (b) ferroptosis, a new type of regulated cell death that is mainly mediated by iron-dependent lipid peroxidation, which is emerging as an important process in valve fibrosis with subsequent calcification [6,7]. Neo-angiogenesis through the TGFβ/Smad pathway is upregulated. Persistent activation of VICs plays a pivotal role as a trigger of valvular calcification. In addition, endothelial impairment has a negative impact in the ability of VECs to maintain normal paracrine signaling, causing disruption of the anti-calcific e-NOS and TGFβ-1 pathways, leading further to osteogenic-like changes [8]. Positron emission tomography (PET) scan studies have shown that inflammation has a lesser role in AS when calcification becomes predominant [9]. This divergence explain in part the atherosclerosis treatments failure in CAS. Finally, in vivo data suggest an association of AS severity with increased levels of circulating intermediate monocytes.

Observational data have shown that cardiovascular risk factors have also been associated to CAS. Dyslipidemia and high Lp(a) are associated with calcification of the AV especially in relatively young individuals, in whom risk is increased 3-fold at an Lp(a) >80th percentile vs. lower levels. Actually, Lp(a) is characterized as an orchestrator of CAS. It enters the aortic tissue at damaged sites and is then converted to lysophosphatidic acid, which in turn triggers the nuclear factor–kB cascade mediating the induction of pro-inflammatory cytokines, TNF, bone morphogenetic NFkB, chemotactic factors, and adhesion molecules, promoting osteogenic mineralization and calcification of the valves through production of alkaline phosphatase (ALP). Analysis of the ASTRONOMER trial revealed that patients with mild to moderate CAS and high Lp(a) levels had faster CAS progression [10]. Moreover, a genome-wide association study by Thanassoulis et al. revealed that the LPA rs10455872 polymorphism was associated with a 2-fold increased risk of AV calcification [11]. Also, Lp(a) is found to accelerate senescence of VECs and production of reactive oxygen species (ROS) in aortic endothelial cells. ROS production further disrupts the integrity of the endothelial barrier by increasing the permeability of the endothelial cell monolayer. Rallidis et al. highlight the importance of Lp(a) in AVC promotion via a ‘three hit’ mechanism including lipid deposition and inflammation [12]. A large-scale genetic study of CAS identified 15 robustly replicated genetic loci, including SORT1–CELSR2, involved in lipid metabolism, and NLRP6, involved in the inflammatory response [11]. The study provided evidence in favor of a causal association for apolipoprotein B, Lp(a), body mass index, and low-density lipoprotein cholesterol.

Arterial hypertension (HTN) and CAS commonly coexist, and the incidence of both conditions are age related. HTN involves pathophysiological mechanisms such as: the upregulation of the renin–angiotensin–aldosterone system (RAAS), endothelium dysfunction, oxidative stress, and activation of the sympathetic nervous system (SNS) and the immune system. Although angiotensin II participates in profibrotic progression of CAS, retrospective studies on RAAS inhibition showed diverging results, as will be discussed later [13].

Diabetes mellitus (DM) augments proinflammatory and tissue factors. AVs obtained from DM patients with severe CAS have a higher expression of C-Reactive Protein (CRP) and are characterized by a 6.6-fold increased accumulation of advanced glycoxidation end products, comparing to non-diabetics. Also, AV from DM patients have been shown to be more mineralized, with high expression of osteogenic markers such as ALP. Hyperglycemia is related with tissue accumulation of advanced glycation end products (AGEs), which are bound by a specific receptor (RAGE) and accelerate calcification of AVs and CAS progression. Nonalcoholic fatty liver disease (NAFLD) is very common in DM type-II. Interestingly, epidemiological studies revealed that NAFLD is closely associated with CAS in both non-diabetic and type 2 diabetic individuals [14].

Ageing is strongly associated with short leukocyte telomere length (LTL), found in CAS, while concurrently the CAS process promotes further the local decrease of TL, establishing a vicious circle. It has been observed that lower bone density associated with ageing promotes vascular calcification by yet not completely explained mechanisms. In addition to their effects on bone density, bisphosphonates have anti-inflammatory and lipid-lowering properties that could prove to be beneficial in patients at risk of developing CAS [15].

As regards gender, data suggest similar progression of CAS severity in both genders. However, it must be noted that for a similar AS severity, females develop more fibrosis, denser connective tissue, and more concentric hypertrophy, while males develop more calcification [16]. Studies have shown that estrogens, independent to Y-chromosome, suppress molecular processes that drive calcification, including repression of receptor activator of nuclear factor kB ligand (RANKL) and NFkB signaling, suppression of NADPH oxidase activity in resident cells and inflammatory infiltrates, and suppression of p53.^16^ Seminal studies showed that post-menopausal estrogen therapies reduce the risk of cardiovascular calcification when administered within the first 5 years of menopause; however, the timing, type, and dosing regimens of estrogen that confer such protection remains an open field of investigation [16].

## 3. Can Diagnostic Data Give Clues to Prevention and Treatment?

In medicine, we like to dichotomize things as black or white, but in nature everything exists as a continuum. Some patients may not quite meet the criteria for severe CAS but still be symptomatic with high BNP levels and signs of advanced cardiac damage [17]. Thus, early recognition of LV impairment is of great importance. Progress has been achieved in the echocardiographic characterization of AV sclerosis [18]. A recent meta-analysis revealed a strong relation between impaired Global Longitudinal Strain and MACE across a wide spectrum of patients with asymptomatic CAS indicating a strong prognostic utility in this population [19]. Cardiac auscultation is neither sensitive nor specific for the diagnosis of CAS. These findings are expected to improve with the use of AI guided stethoscopes. Further advances should be expected with AI contribution.

In CAS, computed tomography (CT) provides the highest-resolution imaging of the AV. Additionally, among all imaging modalities, the CT evaluates best the AV calcification burden. It is suggested that an Agatston score <700AU excluded severe CAS with a high negative predictive value, whereas a score >2000AU suggested severe CAS. Multi-modality imaging techniques, such as combined positron emission tomography (PET)/CT and PET/MRI, are being explored, providing unique insight with respect to valve disease activity, alongside more conventional anatomic assessments of valve and myocardium. Techniques such as 4D-flow MRI for measuring valve EOA have yielded promising results for the future [20]. 18F-NaF PET appears to be a consistent method of detecting early bioprosthetic valve calcification and degeneration, predicting subsequent dysfunction for both TAVR and SAVR.

Discrepancies between various imaging parameters and clinical findings can be reconciled by cardiac catheterization [21]. It can directly measure ΔP, whereas Doppler ultrasound measures indirectly by using the Bernoulli equation. As limitations in the above-mentioned diagnostic tools do exist, novel concepts are needed (Table 1). Learning algorithms and AI technology using ECG and/or image analysis software may minimize interscan variability improving interpretation and diagnosis of CAS in examinations performed even by less-experienced personnel. Recently, deep learning–model for detecting CAS demonstrated high accuracy of significant CAS detection.

## 4. The Importance of Biomarkers

Biomarkers (BMs) reflecting the complex pathophysiology processes have been identified and may complement electrocardiographic an imaging indices of AV changes and LV dysfunction and remodeling (Table 2). Ljungberg et al. found five proteins robustly expressed only in cases with coexistence of coronary artery disease (CAD) but not in patients without, such as: GDF-15, galectin-4, vWF, transferring receptor protein-1, and PCSK-9 [22]. Toutouzas et al. mention more or less the same BMs, stressing their relation to TAVR indications and follow-up [23]. Both groups mention that BMs have a role in indicating which patients should have a TAVR or SAVR and for monitoring the late course. BMs have the potential to identify patients at high risk for specific complications from AVR. Thus, it becomes understandable that the research and upcoming RCTs will shed further light on the role of BMs in risk stratification of patients with CAS (NCT03042104). B-type Natriuretic Peptide (BNP) is increased in the CAS and is the only ΒΜ that is acknowledged by the current ESC guidelines to have a prognostic value [24]. Baseline cardiac troponin levels are predictive of worse outcomes in patients with severe CAS, implying that intervention before the onset of adverse remodeling, or early in its course, may improve prognosis [25]. As regards valve degeneration, BM-related inflammatory processes have also been studied, such as CRP. MDA (Malondialdehyde), a BM of oxidative stress, has been found to increase in patients with AS predicting worse outcome and higher mortality post TAVR. Higher levels of 8-OHdG (8-hydroxy-2-deoxyguanosine) in CAS patients were associated with higher rates of achieving the VARC combined safety endpoint at 30 days post AVR [26]. ST2, a member of the interleukin (IL)-1 receptor family, has been shown to correlate with echocardiographic indices of diastolic dysfunction, predicting symptom onset in patients with severe CAS [27]. In the PROGRESSA study, Lp-PLA2 activity was found to correlate with CAS progression among patients with mild CAS and was very strongly involved in inflammation and subsequently calcification [28]. Kapelouzou and Cokkinos revealed that sclerostin which is locally produced in AV adjacent to areas of calcification is positively correlated with inflammatory factors, oxidative stress and osteoprotegerin expression [29,30]. Other BMs could be miRNAs, such as miR-133a, which predicts the extent of LV hypertrophy reversal post SAVR, miR-21, which was shown to be associated with LV fibrosis, and miR-210, which was independently associated with mortality in patients with moderate-to-severe CAS [31]. BMs previously described mostly involved pathophysiological mechanisms involved in the genesis of CAS or underlying consequential cardiac cell injury. In addition, some BMs may reflect systemic consequences of CAS. Acquired von Willebrand factor (vWF) deficiency, due to the molecular breakdown of the high molecular weight protein vWF in presence of CAS, is one of the most clinically important examples [32]. Proportional to the CAS severity, plasma levels and function of vWF are reduced, which may result in clinically significant bleeding. The combination of gastrointestinal bleeding and CAS is a well-described entity (Heyde’s syndrome). Whether vWF could be used as a surrogate marker of CAS severity remains to be demonstrated. Further efforts towards establishing a set of BMs that can reliably predict CAS progression is urgently needed.

## 5. Management of CAS: Therapy vs. Prevention

Aortic valve replacement (AVR) still represents the only actual treatment for CAS improving survival and quality of life for patients symptomatic with severe CAS. Approximately 150,000 AVRs are performed annually in the USA. However, AVR is a serious operation, especially in older patients. Thus, since the early 2000s, TAVR has been introduced and is steadily gaining ground. The lower morbidity and the shorter hospital stay associated with TAVR has prompted its application to younger patients and those with only moderate symptoms or asymptomatic CAS patients. Since the latest ESC guidelines, based on RCTs and registries, the TAVR indications have been expanded to include patients with a full spectrum of surgical risks [36]. However, an important logistic aspect should not be overlooked. It has been calculated that in the UK more than 200,000 individuals over the age of 70 have CAS, of whom 2/3 would need a very costly TAVR intervention, a fact that would probably wipe out NHS recourses.

### 5.1. Mechanical and Ultrasound Interventions

Ultrasound (US) debridement of the calcific valve was first introduced in the distant 1978. The use of a combination of US frequencies helps to maximize the disruptive effects on dystrophic calcifications of human AV leaflets, and thus can be an effective option for restoration of mobility of the AV cusps especially in elderly patients with very small aortic annulus in which AVR could be extremely difficult [36]. Finally, the application of shockwave technology for CAS concerns the disintegration of the calcific deposits reducing the motion of the leaflets in terminally calcified stenotic valves. This interesting approach was tested in vivo showing promising results regarding biological safety and efficacy without causing large ruptures, thus maintaining tissue integrity [37]. Additionally, small studies using Leaflex™, an alternative novel percutaneous aortic valve repair device for restoring calcified AV leaflet mobility by mechanical scoring, revealed safety and feasibility can improve valve hemodynamics and CAS severity in younger, inoperable, or high-risk cases.

### 5.2. Promising Preventive Therapeutic Drug Targets of CAS

According to the immortal Hippocratic principles, prevention is considered as very important. A better understanding of the complex processes of CAS progression has shifted the field away from a purely degenerative disease model, with more emphasis on active valve mineralization, lipoprotein infiltration, active inflammation, and tissue remodeling. This has led to the identification of multiple possible therapeutic targets, beyond simple correlations between clinical parameters and disease progression. Based on the data, we recommend that a hybrid approach with some commonly used drugs in combination to lifestyle modification could retard CAS progression. In a multifactorial process the use of one single drug alone cannot be expected to be practically fully effective. This has been amply demonstrated in CVD. However, in real-life situations, most RCTs utilize one drug.

#### 5.2.1. RAAS Inhibition

RAAS inhibition is recommended as the main therapy in CAS patients with concomitant HTN. It has been shown that RAAS inhibition has pleiotropic and cholesterol-independent effects that extend beyond afterload reduction slowing CAS progression via improvement of endothelial function and elimination of redox stress, inflammation, and fibrosis [38]. The ARBAS RCT (NCT04913870) will explore whether ARBs inhibit CAS progression and delay myocardial fibrosis. In addition, it has been shown that RAAS inhibitors decrease arterial stiffness, which is usually elevated in CAS, and especially in the acute setting the combination with diuretics prevents adverse outcomes [39].

#### 5.2.2. Hypolipidemic Therapy

Another drug family that has already been tested are statins. In coronary atherosclerosis, statins promote calcification, while decreasing atherosclerotic plaque burden and fibrosis, actions that may explain their lack of influence on CAS progression. In addition, statins lower cholesterol, while increasing PCSK9 levels and LDL-C receptor degradation. Moreover, they do not consistently decrease Lp(a). While there was some promise in a Rosuvastatin trial, this was not validated in large retrospective studies such as SALTIRE (Scottish Aortic Stenosis and Lipid Lowering Trial), SEAS (Simvastatin and Ezetimibe in Aortic Stenosis), or ASTRONOMER [10, 35]. Therefore, current guidelines do not recommend statins for the prevention or treatment of CAS. However, a secondary analysis of the above-mentioned RCTs showed that elevation of Lp(a) and oxidized phospholipids levels is associated with a linear progression of valvular calcification in mild-to-moderate CAS patients and the simvastatin/ezetimibe combination reduced the risk of AVR by 60% in patients with mild but not moderate CAS [35]. In the FOURIER study, PCSK9 inhibition was associated with a lower hazard of new or worsening CAS. Interestingly, Salaun et al. found that high PCSK9 plasma levels were a risk factor for hemodynamic deterioration of surgically implanted biological AVs [40]. PCSK9 may exert an additional role through its interaction with ferroptosis-related genes and complement inactivation, contributing to cell death and dysregulation of cellular tissue homeostasis, thus being another preventive therapeutic target [7]. Recently, several drugs have emerged (Nissen S-SLN 360) which can decrease Lp(a) up to 98% [41]. These new treatments, namely ASOs and siRNA, have a strong Lp(a)-lowering effect. Trials such as the Lp(a) HORIZON Trial (NCT04023552) and the OCEAN (a)-Outcomes Trial (NCT05581303) are currently investigate their clinical efficacy in reducing cardiovascular events.

#### 5.2.3. Antidiabetic Treatment

Maintaining long-term glycemic control as well as the treatment with antidiabetic drugs is beneficial in CAS patients with concomitant DM. This impact might be attributed to the improvement of ventricular loading conditions caused by osmotic natriuresis induced by blocking SGLT2 reabsorption of glucose and afterload caused by a blood pressure reduction and improved vascular function, the improvement of cardiac metabolism, or changes in cytokine production and epicardial adipose tissue mass. Another action of SGLT2 inhibitors is that they can attenuate NLRP3 inflammasome activation and its main product, IL-1-β, in in vivo models supplemented with vitamin D analogues that develop medical vascular calcification [42]. Although, AGEs-RAGE inhibitors seem to be a promising therapeutic target, no data prove this protective effect in CAS patients. Two antidiabetic drugs hold promise: SGLT2 inhibitors which are gaining in use could retard CAS. Recently, it was shown that selective dipeptidyl peptidase (DPP)-4 inhibitors diminish proinflammatory cytokines expression, valvular fibrosis, and calcification of AVs [43]. Also, endothelial-derived NO depletion, as it occurs during CAS progression, enhances DPP-4 expression, thus inducing osteoblastic activation of VICs via accelerated IGF-1 degradation. RCTs, such as the DIP-CAVD trial (NCT04055883), will test whether selective DPP-4 inhibition can alter the progression of CAS. Furthermore, Dapa-TAVI, (NCT04696185) will analyze the benefits of dapagliflozin treatment in patients with severe CAS discharged after TAVR procedure. Furthermore, recent international multicenter data from real heart failure patient population with severe CAS and DM revealed that SGLT2i was showed a favorable left ventricular remodeling effect, diminishing the risk of MACE at 2-year follow-up [44].

#### 5.2.4. Anti-Inflammatory and Antifibrotic Therapies

Since inflammation plays a prominent role in CAS progression, anti-inflammatory agents could be another therapeutic target [33]. However, in vitro data revealed that the already-approved celecoxib, a COX-2 inhibitor, led to hallmarks of myofibroblast activation and calcific nodule formation. Therefore, physicians must carefully use celecoxib and COX inhibitors in elderly patients with risk factors for CAS. Colchicine, via an as-yet incompletely elucidated mechanism, acts as an anti-inflammatory agent [33]. Several trials are running regarding the anti-inflammatory effect of colchicine in CAS progression (NCT05162742). Colchicine may also suppress foam cell formation and cholesterol crystal-induced inflammation, the proliferation of myofibroblasts, smooth muscle cell proliferation, and fibrosis. Recently, the COPE-PCI trial demonstrated reduction of periprocedural myocardial injury. Also, the anti-inflammatory effect of colchicine seems to be beneficial for decreasing the incidence of atrial fibrillation (AF) post-AVR patients and post-PVI/ablation [34]. Also, antagonist of IL-8 receptor2 (CXCR2) and TGFβ signaling could be effective in preventing progression of AV calcification, and in vivo and TLR antagonists, which are already available for clinical use, could be another therapeutic anti-immunity approach [34]. Another drug which has shown promise is Myoinositol Hexaphosphate, which interferes with hydroxyapatite crystal formation. Carbonic anhydrase (CA) which is also involved in bio-mineralization, is associated with atherosclerosis and has been proposed as preventive treatment for CAS [45]. In vivo, acetazolamide (a CA inhibitor) prevented the acidification of leaflets and the regression of CAS [46]. Finally, it was shown that endothelin-1 (ET-1) could be an independent predictor of CAS severity, and tezosentan, an inhibitor of ET-1, might be a new therapeutic therapy to prevent the pathophysiological processes of CAS [47,48].

#### 5.2.5. Antithrombotic Treatment

The valve in native AV valve procedure induces endothelial injury and promote the exposition of prothrombotic factors contained in the valvular tissue such as: activated platelets, inflammatory cells, vWF, TF, and MPs. This intrinsic reservoir of thrombogenic material jailed between the bio-prosthesis and the aortic root is likely related to persistent native AV activity, thrombotic events, and bioprosthetic degeneration post-TAVR. The data support the modulation of the hemostatic system by the pathophysiological course of CAS. In turn, several hemostatic factors are involved in the AV regulation of endothelial dysfunction, inflammation, fibrosis, and calcification during CAS. Given this bidirectional interplay, new therapeutic approaches targeting the hemostatic system within the native AV are required to suppress CAS progression pre-AVR [49]. Oral anticoagulants are the benchmark for preventing thromboembolic complications, especially in conditions such as AF which present in up to 30% of CAS patients. Vitamin K antagonists (VKA), due to interferences with matrix Gla-protein, stimulate cardiovascular calcification and vitamin K2 supplementation is being investigated in the BASIK-2 trial. Koos et al. found that warfarin administration causes widespread valvular and vascular calcification, while Hariri et al. revealed that patients receiving warfarin had a greater degree of CAS compared to NOAC [50]. Recently, it was shown that rivaroxaban reduced cardiac valve calcification compared to VKA [51]. These findings suggest that non-VKA anticoagulants may be considered for patients with CAS requiring long-term oral anticoagulation. Recent data have emphasized the importance of investigating the roles and effects of hemostasis during CAS allowing physicians to adjust antithrombotic treatments post-TAVR. Recently, it was shown that asundexian, a novel orally bioavailable anti-XIa factor, has great antithrombotic efficacy in thrombosis models without bleeding complications. It is probable that factor XIa inhibitors will get attention in the field of interventional cardiology post TAVR, especially for the secondary prevention of SLT and cerebral thromboembolism [52]. Further, in accordance with the current guidelines, all patients who have undergone AVR receiving a bioprosthetic valve must be in pharmacological treatment with ASA. It has been reported that ASA, besides its antiplatelet and anti-atherosclerotic actions, exhibits radical scavenging and antioxidant properties. Therefore, we cannot exclude that ASA treatment could have a protective role against CAS progression or BVF. Of note, the doses used in these studies are supratherapeutic; however, it cannot be excluded that aspirin could be used as adjunctive therapy to other pharmaceutical interventions.

#### 5.2.6. Miscellaneous Candidates

Other candidate drugs driven by physiology could be mineralocorticoid receptor inhibitors, including mitoquinone, a potent anti-oxidant which suppresses calcification, or other antioxidants. Recently, we showed that local delivery of zoledronic acid inhibits CAS and that local drug delivery at AV of healthy pigs and rabbits with a paclitaxel-eluting balloon is safe and effective [53,54,55]. Finally, RESILIA tissue valves, with anti-calcification technology, have demonstrated freedom from structural valve deterioration at 5 years (COMMENCE trial, NCT01757665). Their technological aspects could be used for planning CAS preventive therapies. Antisclerostin antibodies are being tried as a treatment in osteoporotic disease and could be effective in preventing progression of CAS. The calcification paradox implies that treatments for bone diseases might be beneficial for valvular calcification while maintaining bone health. Bisphosphonates and denosumab, a RANKL inhibitor, have an anti-osteoporotic action through modification of calcium homeostasis, reducing CAS progression. Nevertheless, the SALTIRE II RCT did not show a positive effect of denosumab on AV calcification and progression [56]. In addition, bisphosphonates could be beneficial for patients at risk of CAS developing due to their anti-inflammatory and lipid-lowering effects. Also, CAS has been linked to higher levels of parathyroid hormone (PTH) in patients with secondary hyperparathyroidism and renal insufficiency, paving the way for studies evaluating vitamin D supplementation in these patients to prevent CAS progression. Interestingly, in patients with secondary hyperparathyroidism, there is an increased prevalence of LV-hypertrophy, which was reversed after parathyroidectomy and a subsequent reduction of PTH levels, strengthening the notion that the PTH-1R participates in the CAS and hypertrophic mechanism [33]. Finally, circulating extracellular vesicles (EVs) have been recently studied in relation with CV disorders, and there are recent data showing that the high pre-TAVI EV concentration is correlated with five-fold higher odds of adverse outcomes [57].

Studies have shown that post-menopausal estrogen treatments eliminate the risk of cardiovascular calcification when administered within the first five years of menopause; however, the timing, type, and dosing regimens of estrogen that confer such protection remains a very open field of investigation. Additionally, there are data correlating several gene variants (such as VDR, APOE, APOB, IL10, NOTCH1, and ENPP1) with mineralization and CAS; however, these studies are limited by their small sample sizes, and thus more data are needed [58]. Therefore, based on the available data, we should not recommend the use of biological valves in patients with increased pro- and decreased anti-calcific serum factors. Yeghiazaryan et al. published many potential factors associated with early tissue valve degeneration [59]. As more trials on interventional and pharmaceutical trials emerge, their organization, conduction, and evaluation must be ameliorated by artificial intelligence and machine learning. Also, more advanced ways of analyzing results such as network meta-analysis could be employed.

## 6. Conclusions

In regards to current data, we believe that a hybrid strategy with some commonly used drugs in combination with lifestyle modification and mechanical or ultrasound de-calcifying interventions could retard CAS progression. At one step further, the above-mentioned prevention pharmacotherapies combined with “RESILIA-like” devices will be helpful in improving the durability of bioprosthetic valves. Thus, more data will be needed to provide strong evidence that personalized inhibition of specific molecular pathways could also be a therapeutic option for CAS patients. This effort, aided by newer tools such as artificial intelligence or genetic considerations, can evolve more efficiently, thus constituting other example of personalized medicine. Finally, we believe that CAS can be prevented through orchestrated efforts and the employment of newer techniques.

## Figures and Tables

**Table 1:** Pros and cons of various diagnostic imaging modalities of aortic stenosis.

Imaging Modality	Advantages	Limitations
Echocardiography	Main imaging method used to guide AVR Most widely used in clinical practice No radiation exposure Relatively inexpensive	Measures dependent upon LV flow Poor progression to noise ratio Relatively large patient sample sizes required to demonstrate a treatment effect
CT Aortic Valve	Favorable progression to noise ratio Flow independent measurements Relatively small patient sample sizes required to demonstrate a treatment effect	Radiation exposure Ignores the contribution of fibrosis to AS severity and progression
Contrast CT Angiography	Widely available at most centers Flow independent measurements Assesses the burden of both valve calcification and fibrosis	Time consuming image analysis methodology Radiation exposure Requires contrast administration
PET/CT	Assessment of calcification activity High reproducible As an assessment of disease activity may change more quickly than structural or hemodynamic assessments	Available at specialized centers and relatively expensive Radiation exposure Ignores the contribution of fibrosis to AS severity and progression

**Table 2:** Biomarkers reflecting complex pathophysiology processes that can potentially be used in order to diagnose and stratify CAS, and identify patients who are at high risk for complications post AVR.

Biomarker, BMs	Role	References
BNP/NT-pro-BNP	BNP is strong predictor of mortality.	[17,24]
Cardiac Troponins	Baseline troponins are predictive of worse outcome in patients with severe AS.	[25]
Inflammatory BMs	Inflammatory BMs have been positively associated with calcification process.	[33,34]
Oxidative Stress BMs	Oxidative BMs are increased in AS and predictive of worse outcome, including higher mortality post TAVR.	[26,29]
Galectin-3	Galectin-3 has been shown to be an independent predictor of mortality.	[22]
Soluble ST2	sST2 is strong predictor of mortality post AVR.	[27]
Lp(a)	High Lp(a) levels are associated with mineralization and calcification process in aortic valve.	[11,12]
Micro RNA	miR-133a predicts the extent of regression of LV hypertrophy post SAVR, miR-21 is associated with LV fibrosis, and miR-210 is associated with mortality in moderate or severe AS.	[31]
vWF	AS triggers vWF inactivation. Proportional to AS severity, plasma levels and function of vWF are reduced, which may result in significant bleeding.	[32]
TLRs	TLRs are increased in AS, providing a clue for anti-inflammatory therapy.	[33,34]
PCSK9	PCSK9 is increased both in the stenotic valve tissue and serum, providing insight for possible therapeutic options.	[7,22,35]

## Abbreviations

AV: aortic valve; AVR: aortic valve replacement; CAS: calcific aortic stenosis; CT: computed tomography; CVD: cardiovascular disorder; DM: diabetes mellitus; ECM: extracellular matrix; HTN: arterial hypertension; LV: left ventricle; VEC: valve endothelial cells; VIC: valve interstitial cells; SAVR: surgical aortic valve replacement; TAVR: transcatheter aortic valve replacement.
